# A qualitative examination of barriers and solutions to renal transplantation in Malaysia: Key-informants’ perspective

**DOI:** 10.1371/journal.pone.0220411

**Published:** 2019-08-12

**Authors:** Peter Gan Kim Soon, Soo Kun Lim, Sanjay Rampal, Tin Tin Su

**Affiliations:** 1 Department of Social and Preventive Medicine, Faculty of Medicine, University of Malaya, Kuala Lumpur, Malaysia; 2 Nephrology Unit, Department of Medicine, Faculty of Medicine, University of Malaya, Kuala Lumpur, Malaysia; 3 South East Asia Community Observatory (SEACO), Jeffery Cheah School of Medicine and Health Sciences, Monash University Malaysia, Bandar Sunway, Malaysia; Imperial College Healthcare NHS Trust, UNITED KINGDOM

## Abstract

**Introduction:**

End-stage renal disease (ESRD) is increasing globally, and renal transplantation (RT) is the preferred renal replacement therapy to treat ESRD. Internationally, there are only a few countries with RT rates above 50 per million population (pmp), while most of the countries have RT rates between 30–40 pmp. The low- and middle-income countries (LMIC) makes up the majority for the RT rates below 20 pmp in which Malaysia belongs to despite its increasing ESRD rates. There is a need to explore the barriers to access RT with targeted solutions to improve the RT rates and service in LMIC. Thus, a qualitative study was undertaken in Malaysia to address this issue.

**Method:**

A qualitative methodological approach was performed between March-May 2018. Semi-structured interviews were used to explore current RT policy and service availability. Key-informants were identified from a detailed stakeholder analysis of RT system in Malaysia. Interviews were digitally audio-recorded, transcribed verbatim, coded with ATLAS.ti software and underwent thematic analysis thoroughly.

**Results:**

Eight key-informants participated in the study. Barriers and related solutions were classified using the socio-ecological model (SEM). As reported, the barriers and solutions of RT in Malaysia are the results of a complex interplay of personal, cultural, and environmental factors. Key barriers are linked to public’s attitude and perception towards RT and the unaccommodating practices in the healthcare fraternity for RT. Key-informants provided a systematic solution that shed light on how RT could be improved at each SEM level via effective communication, education and inter-agency collaboration.

**Conclusion:**

The SEM provided a framework to foster a better understanding of current practice, barriers, and solutions to RT in Malaysia. This study is the first to explore the barriers and related solutions to RT comprehensively as a whole. Implications of these findings could prompt a policy change for a better RT service delivery model not just for Malaysia but also for other LMIC. Further stakeholder engagement and evaluation of the systems are required to provide insight into best practices that will help to improve the RT rates and service in Malaysia.

## Introduction

The growing disease burden of chronic kidney disease (CKD) had been disregarded until recently when its global mortality rates stood second after HIV/AIDS from its previous 18th place with a considerable increment of 82%.[1] This has led to CKD taking centre stage in health policy reforms because it is significantly linked to the development of end-stage renal disease (ESRD) and cardiovascular disease (CVD) as well as premature mortality.[2–4] The global prevalence of CKD via a systematic review conducted by Hill et al. estimated that 13.4% of the world’s population is inflicted with CKD [5] with about 80% of them occurs in low- and middle-income countries (LMIC).[6] The disproportionatelyhigh burden of CKD in LMIC could be attributed to the greater number of people suffering from diabetes [7, 8] and hypertension [9, 10] in these countries and therefore the increase in CKD incidence. Despite this worrying trend, information on CKD remains inadequate in many LMIC because of varying and inconsistent reporting mechanism used to determine renal failure as well as limited studies that are conducted on CKD [11].

Currently, there are over two million ESRD patients globally [12] who requires renal replacement therapy (RRT) to sustain life, and this number is estimated to rise by 8% annually.[13] It is also estimated that ESRD cases will increase disproportionately in LMIC due to the rising elderly population and their risk of developing non-communicable diseases such as diabetes mellitus and hypertension become more widespread.[2] This would be evident for most LMIC progressing economically and socially and with a significant reduction in infectious disease. The national and global renal registries provide valuable information on ESRD particularly on the socio-demographics, risk factors, treatment modalities, and the outcomes. It is projected that the prevalence of ESRD cases in Malaysia for 2020 and 2040 will be at 51,269 and 106,249 respectively. [14] This could be attributed to the fact that Malaysia is expected to become an aging nation by 2035. [15] In line with the study by Liyanage and colleagues, [2] Malaysia will see a rise in NCD such as diabetes mellitus and hypertension which will cause ESRD to increase exponentially.

Renal transplantation (RT) has emerged as the preferred treatment of choice for ESRD patients when compared to other forms of renal replacement therapy (RRT) like haemodialysis or peritoneal dialysis. Many studies that were conducted either in Malaysia [16] or abroad [17] have demonstrated that RT contributes to a longer survival rate, better quality of life and more economical treatment compared to dialysis.[18–20] Internationally, there are only a few countries with RT rates above 50 per million population (pmp), while most of the countries have RT rates between 30–40 pmp.[12] In most instances, the low- and middle-income countries (LMIC) makes up the majority for RT rates below 20 pmp in which Malaysia belongs to despite its increasing ESRD rates.[12] According to the National Renal Registry of Malaysia, there were a total of 39,711 ESRD patients who were on dialysis in 2016 and out of this cohort, [21] it was estimated that 20,000 patients were eligible for renal transplantation. However, only 82 RT were recorded by the registry during this period.[21] Considering the strong evidence to support RT as the preferred RRT modality, little is known about the reasons behind the plateau of RT rates in Malaysia. With only four transplant centres to provide for RT in Malaysia, [21, 22] there is a gap to indicate a lack of evaluation in the RT system which needs to be addressed. This study was designed to gain an in-depth understanding of the RT knowledge, perceptions, and experiences of key-informants. Fundamentally, we endeavour to address the barriers to access RT, with targeted solutions to improve the rates and service of RT, in Malaysia that may be applicable for other LMIC facing similar challenges.

## Method

### Design and setting

This study was a part of the Access to **Re**nal **TR**ansplantation **A**nd **P**ost-Transplantation **P**rognosis Study of Adults in Malaysia (ReTRAPP). One of ReTRAPP goals is to improve RT rate across Malaysia by investigating patient specific and other factors influencing access to transplantation. The completed ReTRAPP study involved all Malaysian renal transplant recipients as well as key decision makers in the renal transplantation process.

### Participant selection

The initial list of stakeholders in RT was prepared following a review of the National Policies of RT in Malaysia.[22–25] The list of stakeholders was finalized following discussions with senior healthcare personnel involved in RT in Malaysia. The stakeholder analysis (Table 1) adopts the Mendelow’s mapping technique [26] to determine the interest of each stakeholder to improve the RT system and whether they have the authority [27] to do so. Subsequently, a qualitative approach was undertaken with key-informants that maintain a high interest and power in RT (Table 1). Individuals at a national level who has knowledge and experience of improving the RT services in Malaysia were selected purposively to ensure that appropriate informants would provide rich study data.[28, 29] Potential key-informants were invited by email to participate in the study. The sample size was determined by the data obtained, and we sampled to data saturation.[29, 30]

**Table 1:** Stakeholder analysis to determine the key decision makers in renal transplantation.

Stakeholder Analysis	Low Power	High Power
**Low Interest**	Public Private hospitals Insurance companies	Organ donors Media Religious leaders NGO (e.g. National Kidney Foundation) Association of Private Hospital Malaysia Malaysian Society of Anaesthesiologists Malaysian Society of Transplantation Malaysian Medical Association
**High Interest**	Hospitals functioning as transplant centres Clinicians treating RT patients Organ Procurement Team ESRD patients / Renal Transplant Recipients	**National Transplant Council (1 Policy Advisor)** **Transplantation Unit, Ministry of Health (1 Policy Advisor)** **Malaysian Society of Nephrology (2 Clinicians)** **Malaysian Urological Association (2 Clinicians)** **Renal transplant expert / researcher (1 Researcher)** **National Transplant Resource Centre (1 Policy Advisor)**

### Data collection

A standardized semi-structured interview guide was used for all the interviews to explored current RT policy and practices in Malaysia. In-depth one-to-one interviews lasting 50–60 minutes were conducted from March–May 2018. The author used prepared standard interview guide for all key-informants, however, different probing questions were asked to seek clarification and further elaboration from key-informants. Eight interviews were digitally audio-recorded, transcribed verbatim and entered into ATLAS.ti for coding to facilitate data analysis.[31, 32]

### Data analysis

**Fig 1:**
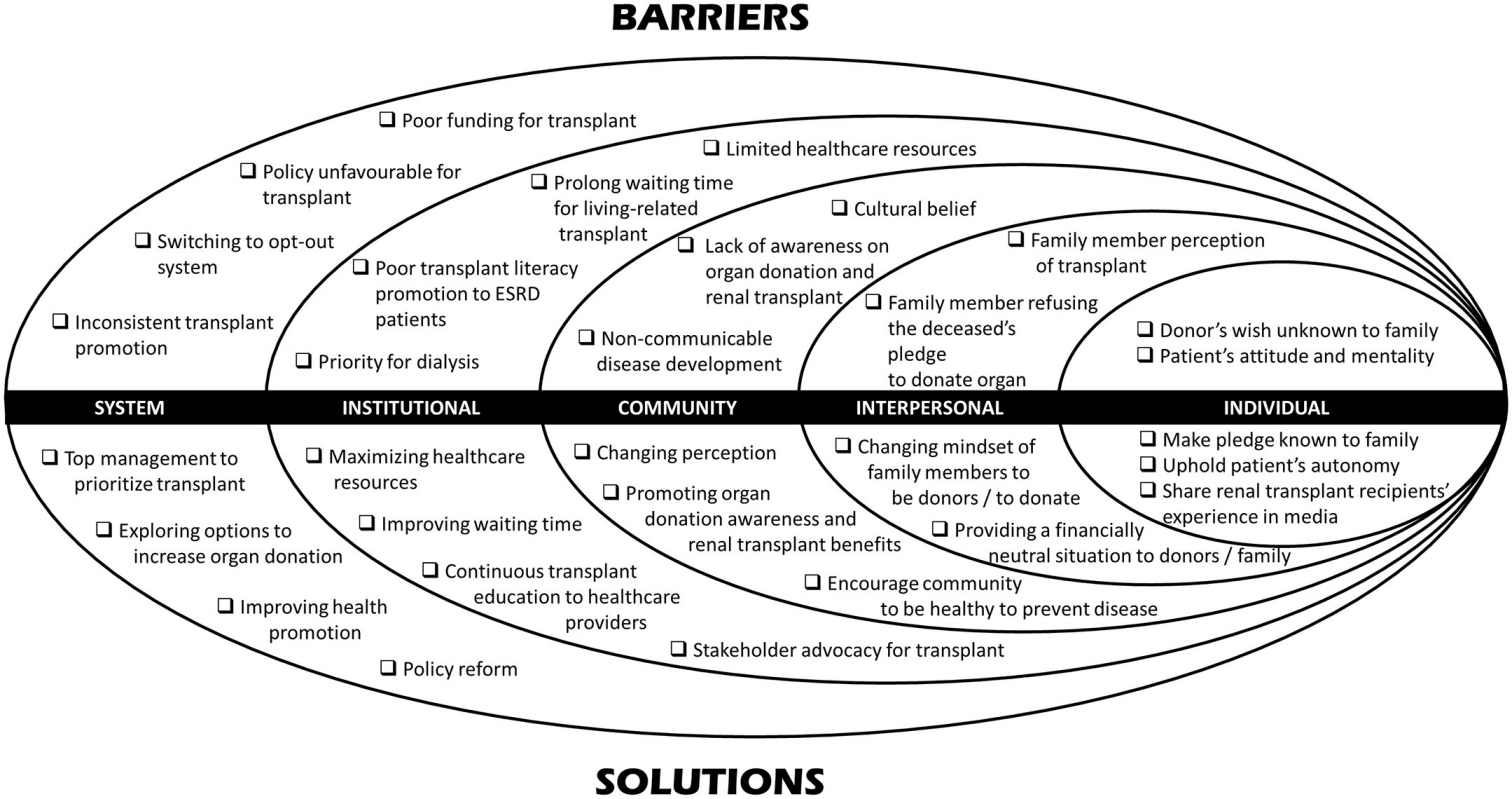
Socio-ecological model presenting barrier and solutions to renal transplantation.

An initial list of thematic codes generated from a subset of the transcripts was developed according to Creswell’s guideline.[30] Themes were identified by perusing through the transcripts. These codes were then modified and refined through thematic analysis approach [33] guided by socio-ecological model to analyse the dynamic interactions across the individual, interpersonal, community, institutional and system levels that could limit the access of RT. Using the socio-ecological model framework as well as local literature [34–38], the results were organized according to key themes that emerged from the data (as summarized in Fig 1). The accuracy of the information provided by key-informants in terms of form and context were contrasted with quantitative data whenever possible.[39] Preliminary analysis was critically explored with other team members to provide a different perspective adding breadth to the topic on RT.[40]

### Ethics

The study received approval by the Medical Research and Ethics Committee of the Ministry of Health Malaysia, Medical Research Ethics Committee of University of Malaya Medical Centre and Research Ethics Committee of Universiti Kebangsaan Malaysia in accordance with the World Medical Association Declaration of Helsinki. During recruitment, all key-informants were provided with an information sheet detailing the research. The interview guide was not shared with the key-informants prior to the interview. Written informed consent were obtained for the interview and the audio recording. Identifiable information was de-identified at the point of transcription and pseudonyms were used for published data extracts to maintain confidentiality.

## Results

A total of eight key-informants were interviewed (5 male, 3 female), including policy advisors, clinicians, and an academic researcher on RT, provideaccount into the RT system in Malaysia. Key-informants described a range of barriers for both living-related and deceased RT that, in their view, prevent the improvement of RT rates in Malaysia but also provided solutions to overcome it. To enhance conclusions to be drawn from the multitude of barriers and solutions identified, themes were grouped into several categories and were matched with the solutions recommended (refer Table 2 for solutions). The findings were organized according to the socio-ecological model for RT adopted from South Africa.[41]

**Table 2:** Possible solution to overcome barriers to RT in Malaysia.

Socio-Ecological Model	KEY Themes	Main Findings as Reported by Key-Informants	Key-informant Quote
**Individual- Level Factor**	Donors to make their pledges known to their next-of-kin	Lack of communication between pledgers and family member. Key-informant suggested that healthcare providers educate pledgers to communicate their intention to donate to family members	*“Those who have actually pledged should tell their family members their desire*. *Then their family members will not go against it*. *So*, *I think those who pledge need to be educated to tell their family members*. *From my experience once*, *the family members know the desire of the deceased beforehand*, *they will not go against it*.*” (male*, *clinician)*
	Healthcare personnel must uphold the patient’s autonomy for organ donation	Key-informant propose that donor’s consent should be final and must not be overruled by their family members	*“If I have made an informed consent to donate*, *can my family stop me*. *If you ask me*, *technically they should not because this is my wish*, *how can they overwrite my wish*. *I could have made it at the time when I am sane*, *when I had all my faculty intact and I was a major*. *So that part of it I think we have to relook at*.*” (male*, *policy advisor)*
	Encourage renal transplant recipients to share their experience of RT on media	Healthy renal transplant recipients should be engaged to share their renal transplant experiences in the media to show the public how well a transplant recipient can be	*“The transplant recipients who were transplanted 35 years ago could have write up done about them in the Malay papers*, *the Chinese papers*, *whatever paper la*. *Put them on television to (tell the public) that Oh My God*!!! *these patients have been living for so many years with a (transplanted) kidney and he is fine*.*” (male*, *policy advisor)*
**Interpersonal-Level Factor**	Ensure that living-related donors are aware their wellbeing is looked after	Key-informant described the process of investigating the donor to ensure the suitability for KI and that the donor’s health will not be in jeopardy	*“(Donor will) go through a lot of test and doctors need to make sure that the donor is protected*, *and we have to be very careful to ensure donor knows their safety is well taken care of*.*” (male*, *clinician)*
	Provide a financially neutral situation for all living donors	The notion of providing free hospital care to living-related donors by the government was reiterated by key-informant as a goodwill for organ donation	*“The government must give incentive for live donor especially kidney donor*, *to waive them from any charges*, *so they don’t have to pay for transplant work-up*, *they don’t have to pay for transplant related hospitalisation*, *surgery and they will be assured of a life-long care*.*”* *(male*, *clinician)*
	Promote awareness among next-of-kin to donate the deceased’s organ	Key-informant opined that the family member’s perception against donating the deceased organ can be solved by providing education and awareness to them	*“I suppose we need to educate them*. *I think that is not a big problem*. *It is for them to accept that the deceased has pledge and follow the desire of that person*.*” (male*, *clinician)*
	Provide a financially neutral situation for all deceased donors	Key-informant stated that public hospitals would waive hospital fees accumulated by the deceased during hospitalization when family member’s consent for organ donation as a token of appreciation	*“We don’t say that we incentivize*, *but we just say that it is one of the ways (we could) ease the burden of the family members*. *Once the family members have donated the deceased organ*. *We will waive the hospital fees*.*” (female*, *policy advisor)*
**Community-Level Factor**	Change perceptions of community on organ donation by collaborating with religious bodies	Key-informant suggested that the Ministry of Health continuously engaged religious authorities in Muslim communities and appointed public figures as organ donor ambassadors to encourage organ donation.	*“We are actively campaigning with JAKIM and also with IKIM (Islamic bodies in Malaysia) in terms of religious perspective and also giving the message to community (about organ donation)*. *We do have organ donation ambassadors to inspire the public*.*” (female*, *policy advisor)*
	Promote organ donation and RT benefits to the younger generation	Key-informant discussed the strategies by Ministry of Health in creating awareness for organ donation by targeting younger generation because they are more receptive of donating their organs	*“*…*strategy for organ donation awareness*, *we have 6 strategies*, *so among the strategy*, *we have one is targeting the younger generation because we believe they’re more receptive and more open towards this cause*.*” (female*, *policy advisor)*
	Encourage public to be healthy in order to prevent renal disease	Key-informant went beyond conventional norms by suggesting that patients with well controlled health indicators would get incentives if they continued to maintain it.	*“*…. *give incentive (to patient) when they are healthy*, *by reducing their annual income tax*. *Then they will go prove to you that their HbA1C (blood sugar level for preceding 3 months) is controlled*, *their blood sugar is controlled*, *and their BMI (Body Mass Index) is controlled*.*” (male*, *policy advisor)*
**Institutional-Level Factor**	Recruit transplant experts from private practice	Experts in renal transplant who previously went into private practice could be engaged for RT in the transplant centre to fill the vacancy of expertise in the field	*“There are probably at least about 10 surgeons*, *nephrologists and intensivists out in the private (practice) who has experience in doing transplant*. *If all of them are included*, *I am sure there will be enough resources*.*” (male*, *clinician)*
	Provide training in RT at renowned transplant hospital	Key-informant considered that training in RT at renowned transplant hospitals in the world would encourage surgeons to take up the sub-speciality.	*“For the past few years we sent our surgeon for kidney transplant training*. *The training mechanism is something like an incentive*.*” (female*, *policy advisor)*
	Centralize the transplant centre for better service	Key-informant suggested that incorporating all the centres into one centralized transplant centre just for transplantation would improve the allocation of resource	*“Just establish one centre first (by) putting all the resources (in one place)*. *Currently it is split in MOH between HKL (Kuala Lumpur Hospital) and Selayang (hospital)*. *Just have one centre*, *where all the resources are placed in one place in an organised department of its own*.*” (male*, *researcher)*
	Improve the supporting services to expedite screening	Key-informant described two mechanism whereby laboratory services can be enhanced; formation of transplant surgery department with lab facilities or use existing facilities but reforming for a shorter pre-transplant assessment.	*“There is a need for a department of transplant surgery (with) immunological lab support*, *pharmacy support for drugs and money to buy all those drugs and all the lab services to support the workup*. *They can actually use existing lab facilities without creating a new lab*, *but they have to have a pathway that is direct*, *responsive and quick*.*” (male*, *researcher)*
	Improve waiting time by cooperating with other departments	Key-informant mentioned that the transplant team coordinate with the pathology or radiology department to improve waiting time for pre-transplant assessment.	*“An understanding between the departments in HKL that allows the patients to be assessed faster which cut down the waiting time for the cardiac assessment and for the radiological assessment*.*” (female*, *clinician)*
	Provide continuous transplant education to healthcare providers	Key-informant proposed that healthcare providers need to be continuous updated on organ donation and transplantation by collaborating with international institute to learn best practices.	*“*… *organizing a sustainable program to educate the transplant fraternity*. *This program is a collaboration between local universities with the worlds′ leading organization in organ donation*, *Donation Transplantation Institute in Spain*.*” (female*, *policy advisor)*
**System-Level Factor**	Require stakeholder to advocate for RT	Key-informant revealed that RT requires many advocates and not just the few who are currently championing it. The dissemination of information by researchers is important to educate the fraternity on the issues faced currently.	*“We need to have more champions because if we rely on one or two loud voice and these loud voice does not reach the ears that supposed to hear it*. *Then you will not going solve this problem*. *If we just discuss about it within these four walls or when you write your research thesis without disseminating it*, *we will continue to discuss about this for many more years to come*.*” (male*, *clinician)*
	Persuade top management in Ministry of Health to prioritize RT	Key-informant lamented that the final decision on funding and prioritization for RT falls to the top management and they need to be prompted on RT importance.	*“*…*what we need is a very firm decision by the ministry of health to agree to this*. *It has to come from the minister downwards*. *The minister has to buy in*, *the DG (Director General) has to buy in*.*” (male*, *policy advisor)*
	Require policy and legislative reform on RT	The revision of the current law governing organ transplant has been in the pipeline and key-informant believed it will help improve the RT	*“We hope that we can table the Bill soon*. *That is one of our effort we also (have) in the pipeline on how to increase the kidney transplant in Malaysia*.*” (male*, *policy advisor)*
	Recruit new pledges for organ donation during vehicle license renewal	Key-informant explored other options on how to implement the idea of focusing organ donation to drivers or motorists by supplementing organ donation form during license renewal.	*“Everyone has to renew driving license*, *and when you apply for your driving license you have to fill up your particulars all these things*. *Some countries actually use that opportunity to seek for donation request*.*” (male*, *clinician)*
	Explore kidney chain donation for endless recipient-donor pairings	Key-informant illustrated the domino effect of kidney donor chain practiced by some foreign country that allows the unrelated organ donation instead of restricting it to living related only.	*“When people pledge*, *they will cause a domino effect*, *the term that they use is never ending altruistic donation; the chain donation*. *For example*, *you come forward to give a kidney to your brother*. *But he cannot proceed to donation because his kidney doesn’t match*. *but I come along*, *and say I’m going to give the kidney to your brother*. *His brother who cannot donate to his brother (ESRD patient) will now donate to another person*.*” (male*, *clinician)*
	Include comprehensive health education and organ donation in school syllabus	The education system needs to incorporate organ donation and transplantation in their syllabus to inculcate altruism and to dispel misconception at an early age	*“I think we need to look into changing the education system to be more inclusive by providing health education as well as teaching about the benefits of organ donation and addressing the stigma the community have on organ donation*.*” (female*, *clinician)*

## Barriers

### Individual-level factor

#### Donor’s wish unknown to next-of-kin

The individual level factor is at the core of the socio-ecological model; key-informants described the patient’s attitude, feeling and decision-making process towards RT. One of the most common problems encountered by healthcare professions is that the deceased donor’s wish to donate is not made known to their next-of-kin.

*“On the other hand*, *can the family donate parts of me (the deceased)*, *without asking me*. *Well*, *if they know your intentions*, *they can*. *How could they know my intention when we never talked about it*?*”*

*(male*, *policy advisor)*

However, when a patient pledge to donate and fail to inform their next-of-kin, it will directly affect their decision not to donate the deceased organs as explained by one key-informant

*“Those who have actually pledged did not tell their family members their desire (to donate their organs)*. *By instinct*, *their family members will not allow the organ to be donated*. *From my experience once*, *the family members may consider donating the organs of the deceased if the desire of the deceased is made know to them beforehand*.*”*

*(male*, *clinician)*

#### Patient’s attitude and mentality

Key-informants who are mainly clinicians interacted with patients and provided feedback that most patients have a hard time accepting their condition

*“When he has renal failure*, *that is not a good time for you to discuss about transplantation because you know that his thought process does not accept it*. *To get him to go and ask someone (family member) for a kidney to give it to him*. *When he says*, *what*? *I don’t even need dialysis*. *At that moment he doesn’t even accept the fact that he needs dialysis*.*”*

*(male*, *clinician)*

Additionally, some of the patients would be indifferent with their condition or treatment leading to a waste of hospital resources

*“Patients will suddenly postpone their operation or delay it because they want to celebrate Raya (Eid) or go on holiday*. *This results in the waste of OT (Operating Theatre) time as well as the work up for the patients which would be invalid after a certain period*.*”*

*(female*, *clinician)*

### Interpersonal-level factor

#### Next-of-kin perception

In the context living-related RT, key-informants understanding of next-of-kin refusal to donate their kidney as a living-related donor was mainly due to fear.

*“The patient (recipient) may wish to have the transplant*. *But the pressure*, *(namely) the physical pressure*, *financial pressure*, *social pressure is on the donor*. *The donor doesn’t get anything (out of the organ donation)*.*”*

*(male*, *clinician)*

One of the key-informant described the burden of living-related donors he had encountered which were not taken into consideration most of the time

*“For some of them*, *the travel is not just from Jalan Ipoh (Ipoh Road) to HKL (Hospital Kuala Lumpur); it may be from east coast coming to HKL*, *it may be Sabah or Sarawak*, *coming to HKL*. *After transplantation*, *they had to stay here for weeks to months to make sure that they’re stable before they go home*. *Where they stay*, *how much money they need for food*, *for accommodation are things that we may not be aware and concerned*.*”*

*(male*, *clinician)*

#### Inconvenience to donor

On top of this, the system is not supportive of the living-related donor and occasionally restrict it as a key-informant recount her experience.

*“*… *the company she worked for did not allow her to take the 6 weeks MC (Medical Certificate) that she was given by the government hospital after the surgery and she had to take unpaid leave*.*”*

*(female*, *clinician)*

#### Refusal to donate

For deceased RT, the next-of-kin refusal to donate the deceased’s organ is one of the major hindrance of organ donation.

*“In Malaysia*, *we are still holding strong culture*, *we are very close knitted family members and without family members’ consensus to decide (if) they want to donate the (deceased) organs*. *It is actually one of the major factors that is actually the major hindrance*. *Family refusal (to donate deceased’s organ)*!*”*

*(female*, *policy advisor)*

Due to the legislative framework on organ donation, next-of-kin have the final consent to donate the deceased’s organs regardless if the deceased has pledged or not

*“The donor’s relative can come and stop at the eleventh hour and say no*, *we refuse to let you take (the organs) although I [deceased] have said you [doctor] can take my parts*, *my wife or somebody can say no I won’t let you take and then you can’t proceed*, *you see*.*”*

*(male*, *policy advisor)*

The reason that most next-of-kin refuse organ donation of the deceased due to the perception of how the deceased will be treated.

“*In my experience*, *most of the relatives assumed that the body would be mutilated if they consent for organ donation*.*”*

*(female*, *clinician)*

### Community-level factor

#### Perception of renal transplantation

The general perception of living-related RT by the public is that it would have serious repercussions on their wellbeing if they donate.

*“Half of the members of public*, *who are in the position to make the decision to donate their organ to their loved one*, *but they do not maybe due to concern for their health and safety*.*”*

*(male*, *policy advisor)*

On the other hand, the public has a strong cultural and religious belief regarding deceased -donation that prevents them from donating because some believed that organ donation is similar to mutilating the dead and it violates the person’s dignity.[42]

*“Most are scared to donate after death as they worry that it may bring repercussions spiritually*…. *Most of them feel that donating organ is something that violate the human body*.*”*

*(female*, *policy advisor)*

#### Lack of awareness of organ donation and RT

The lack of awareness among the public on organ donation stemmed from the delay in promoting organ donation to the community as opined by a key-informant

*“We are actually behind time*. *So of course*, *you cannot expect the rate of pledgers to be so high because we have started very late*. *We should have started a long time before*.*”*

*(male*, *clinician)*

Adding to the lack of awareness, the poor functional health literacy rate in Malaysia that hinders the public from capturing the information on organ donation and renal transplantation is a major problem.

*“But in this country (Malaysia)*, *many are illiterate*. *When I talk about literacy*, *its (means) functionally literate*, *i*.*e*. *they can write and read*. *But how many can understand the implication of the consent and so on*.*”*

*(male*, *researcher)*

Furthermore, the current education system in Malaysia does not include RT into the syllabus compared to other developed countries.

*“There must be a balance of disease prevention awareness and education as well as transplantation and donation education in schools*. *One can never prevent disease 100%*, *hence the answer to saving lives is organ donation and transplantation where possible*. *Unfortunately*, *the current education system does not provide such syllabus*.*”*

*(female*, *policy advisor)*

*“Organ transplantation is not in the local education system*, *because two of my older daughters went to local school and they were no exposure on it*. *My youngest daughter did Cambridge IGCSE* (International General Certificate of Secondary Education) *and there is a section on organ transplantation in their curriculum*.*”*

*(female*, *clinician)*

#### Non-communicable disease development

Most of the key-informants provided similar insight about the community’s unhealthy lifestyle and non-compliance to medication leading to ESRD.

*“It’s due to lifestyle because we know that there is an increase incidence of diabetes*. *So*, *it can be related to that in terms of physical activities which has reduced significantly compared to previously*…. *They think they are getting the complication because of the medicine and not because of the disease*. *So*, *because of this*, *the primary treatment of diabetes and hypertension is so poor*, *in this country*.*”*

*(male*, *clinician)*

### Institutional-level factor

#### Limited healthcare resources

Healthcare resource in terms of human resource, infrastructure, supporting services is the most common limiting factor in improving the RT process in Malaysia. One of the key-informant divulged on the lack of experts in RT.

*“Yes*, *we don’t have many (experts) for transplant*. *Our numbers are very low unfortunately to cater for the whole of Malaysia*.*”*

*(female*, *policy advisor)*

A clinician in the know of the process laments the work burden of the RT team especially the surgeon.

*“The surgeon doing transplant is also the surgeon doing many other things as well*. *So*, *it is extremely exhausting for the individual*.*”*

*(male*, *clinician)*

Under infrastructure, the main facilities in question that were discussed by the key-informants are mainly the intensive care unit [43] and the operating theatre (OT).

*“The limited availability of operating time available for any procedure*. *It’s not necessarily for transplant alone*.*”*

*(male*, *clinician)*

“The current ICU beds that we are using are too close to each other and the ICU does not have isolation area for the transplant patients.”

*(female*, *clinician)*

The supporting services that encompass the laboratory and imaging studies are important in preparing the patients for transplant. But these services are usually inadequate and would delay the RT process.

*“To do a CT (Computerized Tomography) scan*, *to look at normal renal artery for transplant*, *radiology usually will give 2 months appointment*. *To do HLA-typing (Human Leukocyte Antigen) or haplotype typing*, *IMR (Institute for Medical Research) would do once a month*, *(and) sometimes they (would) said no reagent and need to wait for another month*.*”*

*(male*, *researcher)*

#### Prolonged waiting time for living-related RT

The waiting time was partly due to the delay in assessing the patients that were required for the living-related RT as described by the surgeon

*“Because there are so many patients*. *This guy would say*, *we ask for the cardiac appointment*, *they said in six months’ time*, *for a CT scan*, *these guys said*, *appointment in six months’ time*. *So*, *there’s a lot of dilly dally here you are now*.*”*

*(male*, *clinician)*

#### Poor transplant literacy promotion to ESRD patients

The lack of awareness amongst patients could be attributed to the failure of healthcare providers to educate their patients

*“Maybe the doctors are also not doing enough to drum into them*, *you know*, *the importance (of renal transplant)*.*”*

*(male*, *clinician)*

#### Priority for dialysis

The process of providing information on RT is time-consuming. Most healthcare providers are overworked. As such, the nephrologist would likely choose the easier option by suggesting dialysis.

*“The real reason is because to counsel for transplant*, *(it) will takes many hours*. *Repeated counselling*. *Unless you have a dedicated team*, *usually the nephrologist seeing the patients for the very first time*, *they will say dialysis*, *PD (Peritoneal Dialysis)*.*”*

*(male*, *researcher)*

Even funding agencies prefer giving financial support to ESRD patients for dialysis instead of RT.

*“Insurance*, *SOCSO (Social Security Organization) and so on decides on how much money patient gets*, *for what treatment*, *but then there is no policy in those funding agencies to say that we prefer transplant or if you had no contraindication you need to go for transplant*. *But they always have policy for dialysis*.*”*

*(male*, *clinician)*

### System-level factor

#### Poor funding for RT

Funding for RT in Malaysia is minimal compared to other RRT modalities such as dialysis. Key-informants described the propensity to fund dialysis.

*“Yes*, *we need more funding for kidney transplant of course*. *Our budget now pales in comparison to dialysis*.*”*

*(female*, *policy advisor)*

*“The funding of nephrology is mostly channelled to dialysis*. *Recently the government just introduce more subsidy to dialysis in the country which will allow patient to pay only RM10 for each dialysis*.*”*

*(female*, *clinician)*

#### Policy unfavourable towards RT

The key-informant laments that current RT policy has been unchanged since 2007 and request that new revision be made to reflect the current best practice.

*“Our current policy is rather backdated in 2007*. *It urgently needs updates and improvements in all parts of the policy*.*”*

*(female*, *policy advisor)*

Moreover, the current policy does not support RT because there is no political gain even when RT provides a better quality of life and is cheaper to finance.

*“If there is any need to save public money it would be the poorer countries like us*, *but we choose to spend on more expensive dialysis machine*, *incentivise dialysis with the promise of a lower quality of life and lower survival rate compared to a more effective transplant that is cheaper*. *It is more fashionable for people to come to the podium on national television or newspaper to show the dialysis machine*.*”*

*(male*, *clinician)*

#### Inconsistent RT promotion

Promotion of organ donation or renal transplantation programs were conducted inconsistently through the years. The spike in awareness was only temporary when there was news on organ transplantation.

*“Promotion is not effective*. *It is not consistent*. *We do*, *and we stop*. *It’s like what happened a few years ago when we did the first heart transplant*, *we had an increase in the number of people who donate for a brief period*.*”*

*(male*, *clinician)*

#### Switching to an opt-out system

The “opt-in” system currently practised in Malaysia requires that the donors provides informed consent before a person’s organ can be donated.[44] The organ procurement team may proceed to harvest the organs upon the death of the donor or if circumstances allow for procurement. However, the immediate next-of-kin may refuse the organ removal.[39] This would dampen the efforts of the healthcare system to encourage more deceased RT. The change to “opt out” system would increase the organ donation rate but the key-informants feel that the public is not ready.

*“In terms of legislation*, *we cannot go the Singapore way*, *where you have the opt out rule*. *That’s too far in the future*. *We’re not ready for that*. *You cannot introduce an idea which is not ready yet*, *you know*. *People will take offence*, *you know*. *That you’re forcing me to you know*.*”*

*(male*, *clinician)*

*“To decide whether we’re going to do opting in and opting out*, *I think we have to take into consideration of the public’s perception because here in Malaysia we still have a strong cultural belief actually against donation*. *If we were to do opting out system now*, *we’re going to create more resistance and more dissatisfaction from the public*.*”*

*(female*, *policy advisor)*

## Discussion

This research has thematically synthesized evidence of key barriers and solutions perceived by key-informants. It is guided by the socio-ecological model to identify a range of independent and interacting factors that influence RT in Malaysia. It is important to note that only a few studies have examined the interaction of the key elements of the socio-ecological model on renal transplantation. These studies have focussed on racial disparities, [45, 46] medication compliance, [47, 48] transplant education to ESRD patients, [49] renal recipient’s perception of high risk donor [50] and health disparities.[51] To our knowledge, this study is the first to explore the barriers and solutions to RT comprehensively as a whole. The results of this study offer qualitative evidence of the interplay of individual factors, the interpersonal environment, community, the organizational environment and system/policy in practice for Malaysia’s RT process. This highlights the homogeneity of barriers to renal transplantation across diverse healthcare professionals and speaks to a shared understanding of the solutions to overcome them. Our findings show that key-informants were able to identify both barriers and solutions to the RT issues. Consistently, when key-informants identified barriers, they also provided complementary solutions to solve the problems.

Understanding the factors associated with health behaviours has been one of the major challenges for researchers interested in the advance in the treatment of chronic diseases.[52–54] As a result, this research raised questions which must be discussed and included in the context of RT: the perception of the patients regarding RT and the “opting-out” system, and the implication of this to the transplantation service. These findings, in a Malaysian population, match the results of Morgan et al. [55], leading to the conclusion of the existence of questions which are intrinsic to RT, the knowledge, attitude, and values of the patients in accepting RT will impact the routine of the doctors and their role to promote RT. The key-informants strongly believed that doctors who communicated consistently and more often had patients who were more willing to accept the doctor’s choice of RT instead of dialysis.[56] This was validated by Tumin et al. study that demonstrated the role of doctors in advocating for organ donation is highly valued by the public.[35] Therefore, more proactive communication is necessary especially when renal replacement therapy with an emphasis on RT is informed to patients who are diagnosed with ESRD. One way to strengthen communication between doctors and patients would be to provide a standardized training to help improve their competency in communicating and counselling.

In keeping with other studies, [57, 58] the justification of next-of-kin and public to refuse organ donation is mainly due to their fear of organ donation for deceased and living-related RT as well as their cultural and religious belief. The public education system in Malaysia has been shown to be critically inadequate to overcome this barrier of poor organ donation rate.[36] As a method to educate the public, a standardized syllabus on organ donation and transplantation (ODT) would be a good approach to help raise awareness of the younger population.[59, 60] Students who were taught ODT are extremely enthusiastic about it and had more discussions with their families at home, which would indirectly improve the adult’s health literacy.[59] A radical reform to public education of Malaysia would be necessary to incorporate ODT into schools’ curricula as the key-informant suggested. This could be achieved by collaborating with religious authorities, healthcare professionals and community organization to include values and relevant information into the syllabus that support ODT. In Japan, the incorporation of ODT into their national school systems have shown effectiveness, but Akabayashi et al. proposed that more pro-active efforts are essential to involve the school children by using science and technology as a way for teaching and communicating.[61]

When discussing barriers to being present at the hospitals, key-informant suggested the RT services should be centralized to consolidate all services to improve pre-transplant evaluation. Formica et al. showed that by centralizing the pre-transplant workup, besides the significant reduction in the listing time for RT, the economic cost to the centre has also decreased considerably.[62] Likewise, Sultan and colleagues found that the delay in the pre-transplant process is mainly due to the workup being conducted at their local dialysis centres which may not be familiar with the pre-requisite testing resulting in longer waiting time for RT.[63] While addressing these barriers at the hospitals, key-informants suggested that an integrated system for RT service be arranged by coordinating with other supporting services to enhance the pre-transplant evaluation. As the central hub for RT, it would be an appropriate institution to engage experienced surgeons from private practice to train more transplant surgeons in the country. This would allow for future training of highly skilled transplant surgeons, and maintain a high quality and standard of RT practice in Malaysia.[64]

The key-informants emphasized the importance of innovative ideas for recruiting organ donors for RT which would be sustainable. The discussion of incorporating the organ donation registration together with the driver’s license application was proposed by key-informants. This approach is not something new. It has been widely practiced in many countries like the United States of America, [65] United Kingdom, [65] Australia, [66] Canada, [65] New Zealand [65] and South Korea [67]. By partnering with the department of motor vehicles (DMV), it would provide the public with another alternative to register as an organ donor when they apply or renew their driver’s license. Although there are other successful promotional campaigns that focus on workplaces, college campuses and religious institutions in recruiting organs donors, but as Harrison and colleagues indicated, none of these programs was as successful in generating new donors when compared to the DMV campaign.[68] Rodrigue et al. support this finding because half of all organs procured in the United States are authorized through donor registries that administered by DMV with about 42% of all licensed drivers are registered as donors.[69] One reason that the partnership with DMV was successful because it reaches all the drivers who are eligible donors and provide an avenue for immediate registration as donors. To complement this approach, providing incentives to family members of deceased donors as a targeted campaign was suggested by key-informants. As Tumin and colleagues indicate, incentives to family members are highly receptive by the organ donors given the strong family cohesion in the society.[70]

Another approach suggested by the key-informant to mitigate the shortage of organ without converting to the “opting-out” system would be to allow kidney chain donation (KCD) or domino paired donation. KCD provides a solution to a patient who has a willing but incompatible living donor, the patient would receive a compatible kidney from a willing donor who does it out of altruistic reasons.[71] The classical approach of paired kidney donation has been successfully implemented in many countries such as South Korea (since 1991), United States of America (since 1998), Romania (since 2001), Netherlands (since 2004), and United Kingdom together with Australia (since 2007).[72, 73] There are many restrictions that limit the application of this approach such as geographical and legal barriers that Gentry et al. have resolved using innovation such as more variety of participants and relax reciprocity.[74] KCD would not just expand the donor pool but also provide high-quality donor organs, in contrast to organs procured from extended criteria donors and donation after cardiac death donors.[71] There is a need for the transplant community to move beyond the old paradigm so that it will make an impact on the morbidity and mortality of ESRD patients waiting for suitable kidneys. This can be achieved through the cooperation of various transplant centres. Besides that, KCD would positively impact these transplant centres by improving their consistency and fairness in organ allocation. Montgomery and colleagues have demonstrated that KCD has the capacity to enhancing the quality and the number of RT.[75]

### Limitation

While this study provides a solid insight of key-informants’ perceptions of RT in Malaysia, there are some limitations to the study. It is important to note that the findings were only derived from clinicians, policy advisors and a researcher with a focus on RT. But by using these purposive sampling, it ensured that the recruitment of key-informants encompassed the broad cross-section of experts involved in the RT system in Malaysia. The authors acknowledge that while there was a diversity of stakeholders in the RT system, a picture of all potential barriers and solutions was not captured completely because the key-informants interviewed were all doctors and so ReTRAPP was unable to cover the non-healthcare personnel’s perspective on RT.

The principal author who was engaged in conversation with the key-informants may risk introducing biases into the conversation during the interview. Additionally, the influence of the author as an integral part of the interview process brings inherent biases to evaluation and analysis of the information provided cannot therefore be excluded.[29] Although every attempt was made by the author to avoid leading the interviews with the key-informants by having a standard key interview guide for all interviews.

### Recommendation for future research

Specific barriers and solutions highlighted in this research such as the perception towards organ donation and the opt-out system of organ transplantation indicate a need for a rigorous mixed methods study. Following this research, therefore, future research will include more prospective studies exploring other stakeholders’ perception of RT in a Malaysian setting and how targeted interventions improves the RT rates and affect the stakeholders. There is a need for more regional collaborative partnerships and interdisciplinary qualitative research with the aim to address the barriers to access RT in LMIC.

## Conclusion

Malaysia is experiencing a low rate of RT compared to other countries. The use of the socio-ecological model provided a framework to foster a better understanding of the current practice, barriers, and solutions to RT in Malaysia. Evidence from this research has relevance to public health, policymakers as well as doctors who are involved in RT. Guidance for developing culturally competent interventional strategies was elicited from key-informants’ experiences to improve RT rate and services in Malaysia. Implications of these findings may prompt a policy change for a better RT service delivery model not just for Malaysia but also for other LMIC with similar challenges. Further stakeholder engagement and evaluation of the RT system is required to provide further insight into best practices to improve the RT rates and service in Malaysia.

## Supporting information

S1 File ReTRAPP key informant guide. (DOCX)
