# Correction: A qualitative examination of barriers and solutions to renal transplantation in Malaysia: Key-informants’ perspective

**DOI:** 10.1371/journal.pone.0228662

**Published:** 2020-01-29

**Authors:** The PLOS ONE Staff

## Notice of republication

This article was republished on January 16, 2020, to remove a Supporting Information file that was incorrectly included in the originally published article. The article’s Data Availability statement has also been updated to reflect this change. The publisher apologizes for this error. Please download this article again to view the correct version.

## References

[1] Gan Kim Soon P, Lim SK, Rampal S, Su TT (2019) A qualitative examination of barriers and solutions to renal transplantation in Malaysia: Key-informants’ perspective. PLoS ONE 14(8): e0220411 10.1371/journal.pone.0220411 31404075 PMC6690507

