# Supplementary material for: A qualitative examination of barriers and solutions to renal transplantation in Malaysia: Key-informants’ perspective
Source: PLoS One. 2019 Aug 12;14(8):e0220411. doi: 10.1371/journal.pone.0220411 (PMC6690507; doi:10.1371/journal.pone.0220411)
Supplement: S1 File — (DOCX) [file pone.0220411.s001.docx]

**ACCESS TO RENAL TRANSPLANTATION AND POST-TRANSPLANTATION PROGNOSIS (RETRAPP) STUDY OF ADULTS IN MALAYSIA**

**Key Informant Interview Guide**

| **Respondent Name** |  |
| --- | --- |
| **Title** |  |
| **Designation** |  |
| **Organization** |  |
| **Date of interview** |  |

My name is Dr. Peter Gan from the Department of Social and Preventive Medicine, University of Malaya. As you may already be aware, the training of public health physician by the Ministry of Health requires the doctorate candidate to undertake a research study. My research topic is related to the access and outcome of renal transplantation in Malaysia. I hoped that you would be willing to answer a few questions regarding the policy of renal transplantation in Malaysia.

Before we begin, I would like to inform you that your response will be confidential. Nothing you say will be personally attributed to you in any reports that result from this interview. All our reports will be written in a manner that no individual comment can be attributed to a particular person.

Do you have any questions before we begin?

1. Could you share your opinion on rising number of ESRD in the country?

(Probe: NCD issues in Malaysia leading to ESRD)

1. What is the role of renal transplant in treatment of ESRD and what are the challenges?

(Probe: Are ESRD patients receptive of kidney transplantation?)

(Probe: What do you think about the family members understanding of donating their kidney? How about allowing the deceased’s organs to be harvested?)

(Probe: Is the public’s aware of organ donation and transplantation? What are the reasons for the poor public’s reception? Is the promotion and education sufficient?)

1. What are the current programs conducted to promote kidney donation in the country?

(Probe: to ask about deceased and living related kidney transplantation program

1. In your opinion, is the organ donation program effective in increasing the organ donation rate of the country? (total of organ pledges from 1997 – 2015 = 326 738 only ~ 1% of population

(Probe: What are the changes required to improve it?)

(Probe: To ask about living related kidney transplantation and how does it fair in Malaysia)

1. What do you think is the consequences of not the limited availability of kidney for transplant in the country?

(Probe: Do you think overseas kidney transplantation would be an issue?)

1. As a stakeholder, are there any plans to push for an increase in the number of renal transplants conducted in the country?

(Probe: What are the plans to promote renal transplant to patients, family members, the community and the healthcare professionals?)

(Probe: To ask about the opting out system)

1. Do you think whether the current policy and practice on renal transplantation need to be revised?

(IF YES, ASK:) how and which direction do you see it going?

what about the feasibility of the current policy?

What are the opportunities do you see with the current policy?

(IF NO, ASK:) Why do you think so?

1. What challenges do you think the renal transplantation program in Malaysia is facing and how to overcome it?

(Probe: What are the limitations in the hospitals dealing with kidney transplantation?)

(Probe: What’s keeping the renal transplantation program from expanding? If participant mentions resources, use as a probe: There probably were times when there was resource, but things didn’t happen. What was going on there? Why didn’t things happen?)

(Probe: One stop transplant centre and pre-emptive transplant)

1. What needs to happen to help the policymakers to take the lead in addressing these challenges?

(Probe: Think back to the challenges we talked about in #2. What could be done about those?)
